# Does Tobacco Smoking Affect the Postoperative Outcome of MIS Lumbar Decompression Surgery?

**DOI:** 10.3390/jcm12093292

**Published:** 2023-05-05

**Authors:** Morsi Khashan, Dror Ofir, Uri Hochberg, Haggai Schermann, Gilad J. Regev, Zvi Lidar, Khalil Salame

**Affiliations:** Tel Aviv Sourasky Medical Center, Sackler Faculty of Medicine, Tel Aviv University, Tel Aviv 6423906, Israel

**Keywords:** smoking, spine surgery, minimally invasive, decompression, radiculopathy

## Abstract

Background: Tobacco smoking is a major cause of morbidity and mortality worldwide. Several authors reported a significant negative impact of smoking on the outcome of spinal surgeries. However, comparative studies on the effect of smoking on the outcome of minimally invasive (MIS) spinal decompression are rare with conflicting results. In this study, we aimed to evaluate clinical outcomes and postoperative complications following MIS decompression in current and former smoking patients compared to those of non-smoking patients. Methods: We used our prospectively collected database to retrospectively analyse the records of 188 consecutive patients treated with MIS lumbar decompression at our institution between November 2013 and July 2017. Patients were divided into groups of smokers (S), previous smokers (PS) and non-smokers (N). The S group and the PS group comprised 31 and 40 patients, respectively. The N group included 117 patients. The outcome measures included perioperative complications, revision surgery and length of stay. Patient-reported outcome measures included a visual analogue scale (VAS) for back pain and leg pain, as well as the Oswestry disability index (ODI) for evaluating functional outcomes. Results: Demographic variables, comorbidity and other preoperative variables were comparable between the three groups. A comparison of perioperative complications and revision surgery rates showed no significant difference between the groups. All groups showed significant improvement in their ODI and VAS scores at 12 and 24 months following surgery. As shown by a multivariate analysis, current smokers had lower chances of improvement, exceeding the minimal clinical important difference (MCID) in ODI and VAS for leg pain at 12 months but not 24 months postoperatively. Conclusions: Our findings show that except for a possible delay in improvement in leg pain and disability, tobacco smoking has no substantial adverse impact on complications and revision rates following MIS spinal decompressions.

## 1. Introduction

Tobacco smoking is still a major cause of morbidity and mortality worldwide. According to the WHO, in 2019 about 20% of the world’s population were smokers. Smoking was responsible for 8 million deaths in 2019 and about 200 million deaths over the last 30 years [1,2]. The main disease conditions associated with tobacco smoking include cardiovascular, respiratory, cerebrovascular and peripheral vascular disease in addition to cancer and impaired immunity.

Data from the American College of Surgeons National Quality Improvement on surgical patients from different disciplines revealed that smokers had increased postoperative morbidity and mortality [3]. Smoking is believed to have a negative effect on the outcome of lumbar and cervical spine surgery with an increased risk of perioperative complications including wound complications [4,5,6,7].

In the last decade, minimally invasive (MIS) spine surgery has seen increasing popularity. Evidence shows comparable long-term outcomes of MIS spinal decompression and traditional open decompression procedures. However, MIS reduced infection rates, soft tissue injury and intraoperative bleeding [8,9,10,11,12,13,14]. The smaller surgical wounds and the preservation of soft tissue in MIS decompression may therefore negate the proven negative effect of smoking on outcomes and specifically that on wound complications, following open spinal decompression.

Scientific data on the influence of tobacco smoking on minimally invasive lumbar spinal decompression are limited with conflicting results. In two recently published studies, one [15] showed that smoking was not an independent predictor of any patient-reported outcome measures (PROMS), while the other [16] found that smokers had inferior outcomes and lower odds of reaching meaningful clinically important differences (MCID). Therefore, we aimed to evaluate the patient-related outcome, postoperative complications and revision rates following minimally invasive spinal decompression in current smokers, former smokers and those who have never smoked. 

## 2. Materials and Methods 

The current research received approval from the IRB of the authors’ affiliated institution. We retrospectively reviewed the prospectively collected data of all the patients who underwent minimally invasive lumbar decompression at our institute between November 2013 and July 2017. We included patients older than 18 years who underwent elective minimally invasive discectomy or laminoforaminotomy for degenerative lumbar disease, with a minimal follow-up period of 24 months. All patients suffered preoperatively from radiculopathy or neurogenic claudication with or without back pain and had failed conservative treatment which included non-steroidal anti-inflammatory drugs, epidural steroid injections and physiotherapy. The exclusion criteria included patients operated on at more than 2 spinal levels, patients with non-degenerative spine pathology and patients who had undergone procedures that included spinal instrumentation or non-instrumented fusion. Smoking patients were defined as patients who smoked at the time of surgery or patients who had stopped smoking less than six months before surgery. Past smokers were defined as patients who had quitted smoking six months or longer before the surgery. Smoking patients were assigned to the S group, former smokers who had quitted smoking were assigned to the PS group and non-smoking patients were assigned to the (N) group. 

MIS decompression procedures were performed routinely under general anaesthesia using a 20 mm tubular retractor system (METRx, Medtronic Sofamor Danek, Memphis, TN, USA) and a surgical microscope. Surgery was performed using a unilateral approach with either ipsilateral or bilateral canal decompression, as described previously [17,18].

Demographic variables and preoperative data, including medical history, comorbidities and American Society of Anaesthesiologists (ASA) score, were recorded. To simplify the medical history data presentation, we classified patients’ comorbidities into 10 categories: cardiac, vascular, endocrine, metabolic, neoplastic, gastrointestinal, neurological, pulmonary, renal and connective tissue diseases. We used the number of pack years as an estimate of the lifetime tobacco smoking exposure for current and past smokers. This was calculated by multiplying the number of cigarette packs smoked daily by the number of smoking years. 

Outcome measures included length of hospital stay (LOS), intraoperative and postoperative complications, revision surgery and patient-reported outcomes that were collected using questionnaires filled during routine outpatient visits. Patient-reported outcomes included the Oswestry disability index (ODI) and the visual analogue scale (VAS) for back and leg pain, before surgery, and one and two years following surgery. A clinically significant improvement in disability and pain was defined as any improvement that exceeded the minimum clinically important difference (MCID) for ODI and VAS. We used previously published MCID values as follows: 1.16 for NRS back pain, 1.36 for NRS leg pain and 12.40 for ODI [19].

In order to control for potential confounders, multivariate logistic regression was performed to identify risk factors for surgical site infection, revision surgery and prolonged hospital stay. We also used this analysis to identify predictors for improvement exceeding the MCID in each of the patients’ reported outcome measures. Smoking status and the number of pack years were included in this analysis in addition to age, BMI above 30 and diabetes, all of which were previously shown to influence the outcome of MIS spinal decompression. For the statistical analysis, differences between the groups were determined using one-way ANOVA test for continuous variables. The X^2^ test and the Fisher exact test were used to evaluate and compare categorical variables. Preoperative to postoperative ODI and VAS changes within each group were analysed with paired t-tests. A *p*-value < 0.05 was considered statistically significant. Multivariate logistic regression was applied to evaluate the effect of smoking on selected outcomes while adjusting for possible confounders. None of the baseline variables were significantly different between the study groups. Therefore, we step-wisely included those variables that were biologically plausible predictors of outcomes and were relatively prevalent in the study group. First, we adjusted for pack-year data, which complemented smoking status. In addition, we included age, diabetes and obesity (BMI above 30 kg/m^2^). The inclusion of gender did not contribute significantly to model accuracy and it was therefore omitted to avoid overfitting. Analysis was performed in the R statistical program, R Core Team (2021). R is a language and environment for statistical computing (R Foundation for Statistical Computing, Vienna, Austria). We also used SPSS version 28.0.1.1 (IBM Corp., Armonk, NY, USA).

## 3. Results

One hundred and eighty-eight patients, (55% males) met the inclusion criteria and were divided into three groups. The S group comprised 31 smoking patients, the PS group included 40 former smokers who had quitted smoking and the N group included 117 patients who had never smoked. The minimum follow-up period was 24 months and the average was 37.6 ± 7.8 months. The average age was 59.6 ± 17.5 years and it did not differ significantly between the groups (*p*-value = 0.78). The prevalence of diabetes mellitus was higher in the smoking group (*p*-value = 0.04). Other comorbidities and the distribution of ASA scores were comparable between the groups. Overall, 103 (54.7%) patients underwent discectomy, and 85 (45.3%) patients underwent laminectomy with no significant difference in the distribution of the surgery type between groups. The vast majority of patients underwent one-level decompression. Only a few patients underwent two-level decompression and the proportion of them was similar between the groups (*p*-value = 0.39). The average number of pack years for the current and past smokers was 31.1 ± 28 and did not differ significantly between the groups (Table 1).

The overall complications rate was 14.9 % and was higher in non-smokers compared to current and previous smokers but did not reach statistical significance. All recorded complication rates were comparable between the groups and did not differ significantly. Incidental durotomy occurred in 15 (7.9 %) patients, most of whom were in the N group (9%) while the S group had the least number of patients undergoing this (2%). Two patients from the N group had a transient motor deficit. Residual canal stenosis was encountered in three patients (one in each group). Recurrent disc herniation occurred in six patients, three (2.5%) of whom were in the S group and three of (4.9%) whom were in the N group. One patient from the PS group had a superficial surgical site infection (Table 2).

Ten patients (5.3%) underwent revision surgery. The revision rate was 9.7% in the S group compared to 4% in the N group and 5% in the PS group (*p*-value = 0.39) (Table 2). In the N group, one patient needed revision discectomy for recurrent disc herniation at the index level (39 months postoperatively) and one patient was operated on again 30 months after the index surgery for adjacent level stenosis. Three patients underwent transforaminal lumbar interbody fusion (TLIF) at the same operated level due to mechanical back pain in one patient (18 months postoperation), mechanical back pain and radiculopathy due to foraminal stenosis in the second patient (seven months postoperation) and due to back pain and progression of pre-existing grade I degenerative spondylolisthesis in the third patient (17 months postoperation). Among the current smokers, one patient required revision discectomy due to disc re-herniation (18 months postoperation) and one patient needed decompression for residual stenosis at the same operated level (29 months postoperation). TLIF at the index level was performed in a third patient for radiculopathy and back pain due to degenerative spondylolisthesis with foraminal stenosis on MRI without a change in spondylolisthesis (30 months postoperation). In the PS group, wound revision was carried out in one patient three weeks postoperatively due to infection of the surgical site. Another patient underwent revision decompression due to re-stenosis at the index level (23 months after the index surgery).

The average ODI score and VAS score for back and leg pain at the baseline were comparable between the groups (Table 3). At one year and two years after surgery, there were no significant differences between the groups in terms of ODI score or VAS score for back pain. Both ODI and VAS scores for back pain increased mildly at two years compared to one year following surgery; however, this re-increase did not reach statistical significance. The VAS scores for leg pain were also comparable between the groups at baseline and one year and two years postoperatively. All groups showed significant improvement in the ODI and VAS scores for back and leg pain at one and two years post-surgery compared to the baseline (Table 3). In order to evaluate the clinical significance of the improvement in pain and quality of life, we compared the proportion of patients with improvement in achieving the MCID for each score at 12 and 24 months postoperatively (Table 4). These proportions were comparable between the groups in the ODI and VAS scores for back pain. Looking at VAS scores for leg pain, a significantly lower proportion of patients with clinically significant improvement was found in the group of current smokers compared to the groups of former smokers and non-smokers (*p* value = 0.02) at one year postoperatively. These results suggest that compared to the other groups, a smaller proportion of smoker patients achieved clinically significant improvement in leg pain at the one-year follow up, but the difference was not significant two years after surgery. 

In multivariate analysis, we identified the number of pack years and a BMI above 30 as risk factors for surgical site infection (*p*-value = 0.02 for each). A BMI above 30 increased the odds of infection by 3% and every additional pack year increased the odds of infection by 0.1%. Older age was significantly associated with a LOS longer than 24 h (*p*-value = 0.02). Current smokers and older patients had lower odds of having an improvement exceeding the MCID in ODI scores at one year postoperatively (*p*-value = 0.02 and 0.04, respectively). Interestingly no such association was found at two years postoperatively. Current smokers had significantly lower odds (odds ratio = 0.79; *p*-value = 0.04) of having an improvement exceeding the MCID in VAS scores for leg pain at one year postoperatively. Once again, no such association was found at two years postoperatively. 

## 4. Discussion

In this study, we compared the outcome and complication rates of MIS decompression between current smokers (S group), previous smokers (PS) and non-smokers (N). Results were compared at one and two years following surgery. The three groups were comparable in their preoperative variables including their BMI and ASA scores, except for the higher proportion of patients with diabetes mellitus in the S group. The patients’ reported outcome measures improved significantly in all three groups. Current and previous smoking did not affect the complications or revision surgery rates following MIS lumbar decompression. 

Previous studies showed a negative impact of tobacco smoking on the clinical outcome and complication rate in a range of surgical disciplines [3,20]. Smoking was found to be associated with higher complication rates and less improvement following conventional spine surgery [4,5,6,7,21]. Nevertheless, research on the influence of tobacco smoking on the outcome following minimally invasive spine surgery is limited and the results are controversial [15,16].

Patients’ reported outcomes: We found significant improvement in the ODI and VAS scores for back and leg pain in all three groups after one year (*p*-value < 0.01) and after one years (*p*-value < 0.01). We noted a slight increase in ODI and VAS scores for back pain after two years compared to the scores at one year following surgery, but the re-increase did not reach statistical significance. These findings suggest that MIS decompression results in improvement in pain and disability at one and two years following surgery in both smoking and non-smoking patients. We found that the odds of achieving improvement that exceeds the MCID two years postoperatively were not significantly different between current smokers, previous smokers and non-smokers. However, looking at VAS scores for leg pain, we found that at 12 months, a lower proportion of smoking patients achieved an improvement exceeding the MCID compared to the other groups.

In the multivariate analysis, we found that current smokers and older patients had lower odds of having improvement exceeding the MCID in ODI scores at one year but not at two years postoperatively, suggesting that in these patients, clinically significant improvement was delayed compared that in the rest of our cohort. This difference did not reach significance two years following surgery. Similarly, in the multivariate analysis, current smokers had significantly lower odds (odds ratio = 0.79; *p*-value = 0.04) of having improvement exceeding the MCID in VAS scores for leg pain at one year postoperatively. Once again, no such association was found at two years postoperatively. These findings indicate a possible delayed improvement in leg pain and disability in current smokers.

Two recent studies evaluated the influence of smoking on postoperative outcomes of minimally invasive lumbar decompression with conflicting results [15,16]. Beck et al. compared the functional outcomes and meaningful clinically important difference (MCID) between 25 smokers and 70 matched non-smokers one year after lumbar tubular micro-decompression and found inferior outcomes and an inferior MCID in the smoking group. In contrast, our results are in agreement with those of the study by Goyal et al. which included 195 patients (22 current smokers, 52 former smokers and 121 non-smokers) and revealed that smoking status did not influence any patient-reported outcome measure. In the latter study, 68 of the patients were operated on using minimally invasive techniques and the others were operated on using the conventional approach. 

**Length of stay**: A total of 100 of our patients (53%) were discharged within 24 h and 154 patients (82%) were discharged within 48 h from surgery. The mean LOS was found to be comparable between the groups (Table 2). These results imply that smoking has no impact on the length of stay following MIS decompression and are in agreement with the results from the Norwegian registry [21]. Interestingly, in the multivariate analysis, it was found that older age was associated with a length of hospital stay longer than 24 h. This finding is consistent with that of our previous study investigating the impact of age on the outcome of MIS decompression [22].

**Complications:** Incidental durotomy rates were comparable between the groups (*p*-value = 0.44), being 7.9% in the whole cohort. Previous studies reported similar rates of incidental durotomy in open lumbar decompression procedures [23] as well as in minimally invasive decompression of the lumbar spine [24]. Dural tears were treated using the same tubular approach. Sutures and designated patch sealants were used. No cases in this report were converted into an open approach due to incidental durotomies.

Two patients in the entire cohort (0.01%), both from the control group, had a radicular motor deficit after the surgery with complete recovery within a few days after the surgery. In previous studies of general spinal surgery, new neurological deficits occurred in 0.12% of 11,817 patients and 0.18% of 12,375 patients [25,26]. 

One patient from the PS group had a superficial wound infection. This is consistent with multiple studies that demonstrated a very low infection rate in MIS decompression surgeries. Moreover, comparative studies found significantly lower rates of surgical site infection in MIS spinal surgeries compared to open procedures [27,28]. While tobacco use was identified as a risk predictor for surgical site infection after spinal surgery in some reports [29], other studies failed to demonstrate a causative relationship between smoking and surgical site infection [30,31]. It is probable that we could not show differences in the infection rate between the groups due to a low infection rate in the MIS spine procedure. Such a low infection rate will necessitate a much larger cohort in order for differences to be demonstrated between the groups. In a multivariate analysis, we identified the number of pack years and a BMI above 30 as risk factors for surgical site infection. However, our study’s ability to detect differences in infection rates was limited and we advise careful interpretation of this finding.

**Surgical revision:** The revision rate in this series was 5.3% and was not different between the three groups. Goyal et al. [15] reported a revision rate of 3.6% within 90 days after surgery, which was not affected by the patient’s smoking status. In a series of 500 patients following an open laminectomy, Bydon et al. identified smoking as the strongest predictor of reoperation [32]. The revision rate in our series is consistent with the rates reported in previous studies on minimally invasive tubular procedures, which were between 1.2 and 15% [24,33] (Table 5).

## 5. How Does MIS Decompression Prevent the Negative Impact of Tobacco Smoking?

In open posterior spine surgery, the long incision together with the extensive dissection of muscles from the posterior bony elements and continuous self-retaining retraction result in significant oedema of the paraspinal muscles, leading to significant soft tissue damage, denervation and eventually to the reduction in their cross-sectional area [34,35,36]. By preventing these effects, MIS spinal procedures enable the better preservation of soft tissue and promote the healing process after surgery. In previous studies, we found that old age [22] and diabetes mellitus [37], two variables which impose a deleterious effect on the outcome of open spine surgery, had no similar influence in patients undergoing minimally invasive surgery. 

Smoking has been shown to interfere with the healing of wounds, soft tissues and bone through the vasoactive effect of nicotine on peripheral tissue blood flow and oxygenation, as well as on the attenuation of inflammatory cell infiltration in the wound [4,34,35]. In addition, on the molecular level, nicotine inhibits the gene expression of the fibroblast growth factor, vascular endothelial growth factors and bone morphogenic proteins [38]. This attenuates epidermal regeneration and neovascularization and reduces collagen synthesis and the deposition of mature collagen. By reducing the incision length and soft tissue injury, MIS decompression procedures may minimize the adverse effects of nicotine consumption, thus allowing comparable results between smoking and no-smoking patients (Table 6). 

## 6. Limitations

Our study has a few limitations that are similar to those of the above-referenced works [15,16]. First, this study was a retrospective analysis based on prospectively collected data. Final conclusions about the impact of smoking on MIS spinal decompression will better be supported by prospective studies. Another limitation is the small number of patients; a larger cohort could add to the power of the statistical analysis and could allow a demonstrations of more differences in outcomes between the groups. 

**Conclusions:** Our findings show that except for a possible delay in the improvement in leg pain and disability, tobacco smoking has no substantial adverse impact on complications and revision rates following MIS spinal decompression. Future prospective studies with larger cohorts are needed to confirm and support our results.

## Figures and Tables

**Table 1:** Demographic and preoperative variables—comparison between the groups.

		N	S	PS	P
Total		117	31	40	
Age		60.4 ± 17.9	59.6 ± 14.9	57.1 ± 18.2	0.19
BMI		27.9 ± 3.7	25.3 ± 6.2	26.4 ± 6.1	0.59
Male		58 (49.6%)	19 (61.3%)	26 (65%)	0.18
Discectomy		63 (53.8%)	18 (58.1%)	22 (55%)	
Decompression		54 (46.2%)	13 (41.9%)	18 (45%)	0.91
number of levels	One	7 (5.7%)	5 (12.2%)	2(8%)	
	Two	115 (94%)	36 (88%)	23 (92%)	0.39
ASA score	I	28 (23.9%)	4 (12.9%)	9 (22.5%)	
	II	51 (43.6%)	22 (71%)	23 (57.5%)	
	III	33 (28.2%)	5 (16.1%)	6 (15%)	
	IV	5 (4.3%)	0 (0%)	2 (5%)	0.14
Comorbidities	Cerebrovascular	9 (7.7%)	1 (3.2%)	1 (2.5%)	0.55
	Renal	6 (5.1%)	1 (3.2%)	1 (2.5%)	0.88
	Oncological	12 (10.3%)	0 (0%)	4 (10%)	0.15
	HTN	52 (44.4%)	17 (54.8%)	18 (45%)	0.57
	DM	30 (25.6%)	11 (35.5%)	8 (20%)	0.04
	Cardiovascular	54 (46.2%)	14 (45.2%)	17 (42.5%)	0.96
	Pulmonary	5 (4.3%)	3 (9.7%)	2 (5%)	0.43
	Endocrine	15 (12.8%)	4 (12.9%)	6 (15%)	0.95
Pack years			29.6 ± 22.2	33 ± 35.1	0.1

N—non-smokers, S—smokers, PS—previous smokers.

**Table 2:** Length of stay, complications and revision surgeries—comparison between the three groups.

		N	S	PS	P
Total		117	31	40	
LOS		2.5 ± 5.7	2.2 ± 4.1	2.2 ± 3.4	0.887
Complications	Total	21 (17.9%)	3 (9.7%)	4 (10%)	0.43
	Immediate complication	13 (11.1%)	0 (0%)	4 (10%)	0.14
	Complications after discharge	11 (9.4%)	2 (6.5%)	0 (0%)	0.29
	Durotomy	11 (9.4%)	0 (0%)	4 (10%)	0.18
	Neurological	2 (1.7%)	0 (0%)	0 (0%)	0.72
	SSI	0 (0%)	0 (0%)	1 (2.5%)	0.38
	Pneumonia	1 (0.9%)	0 (0%)	0 (0%)	0.65
	UTI	0 (0%)	0 (0%)	0 (0%)	-
	PE/DVT	0 (0%)	0 (0%)	0 (0%)	-
	Recurrent disc herniation	3 (2.6%)	3 (9.7%)	0 (0%)	0.23
	Residual stenosis	1 (0.9%)	1 (3.2%)	1 (2.5%)	0.28
	Other complications	2 (1.7%)	0 (0%)	0 (0%)	0.72
Revision		5 (4.3%)	3 (9.7%)	2 (5%)	0.51

SSI—surgical site infection, UTI—urinary tract infection, PE—pulmonary embolism, DVT—deep vein thrombosis, N—non-smokers, S—smokers, and PS—previous smokers.

**Table 3:** Comparison between the mean values of ODI and VAS for back and leg pain between the three groups and within each of the three groups at the baseline, one and two years postoperatively.

		Baseline	1 Year	*p*-Value (1 Year-Baseline)	2 Year	*p*-Value (2 Years-Baseline)
ODI	All patient	51.8 ± 19.8	19.1 ± 24.8	<0.001	25.6 ± 26.0	<0.001
	N group	52.8 ± 18.5	19.2 ± 24.4	<0.001	25.2 ± 25.1	<0.001
	S group	53.0 ± 17.8	22.4 ± 28.8	<0.001	28.7 ± 30.1	<0.001
	PS group	48.0 ± 24.7	16.6 ± 22.8	<0.001	24.7 ± 25.8	<0.001
*p*-value between the groups		0.38	0.62		0.78	
VAS Back pain	All patient	6.4 ± 3.3	2.6 ± 3.2	<0.001	3.3 ± 3.5	<0.001
	N group	6.5 ± 3.2	2.6 ± 3.1	<0.001	3.3 ± 3.4	<0.001
	S group	6.4 ± 3.3	3.3 ± 3.6	<0.001	3.5 ± 3.8	0.003
	PS group	6.1 ± 3.3	2.0 ± 3.0	<0.001	3.3 ± 3.6	0.003
*p*-value between the groups		0.78	0.2		0.97	
VAS leg pain	All patient	7.5 ± 2.9	2.3 ± 3.2	<0.001	2.0 ± 3.3	<0.001
	N group	7.8 ± 2.6	2.2 ± 3.2	<0.001	2.0 ± 3.5	<0.001
	S group	6.5 ± 3.4	3.4 ± 3.7	<0.001	1.3 ± 2.6	<0.001
	PS group	7.4 ± 3.0	1.8 ± 2.7	<0.001	2.3 ± 3.0	<0.001
*p*-value between the groups		0.09	0.1		0.4	

ODI—Oswestry disability index, VAS—visual analogue scale, N—non-smokers, S—smokers, and PS—previous smokers.

**Table 4:** Comparison of the proportion of patients in each group with improvements exceeding the minimal clinical important difference (MCID) at the baseline, one and two years postoperatively.

		N	S	PS	Total	*p*-Value
ODI 1 year	Patients with available scores	114	28	37	179	
	Improvement above the MCID	68 (60%)	12 (43%)	20 (54%)	100 (56%)	0.27
ODI 2 years	Patients with available scores	94	21	35	150	
	Improvement above the MCID	56 (60%)	10 (48%)	19 (54%)	85 (57%)	0.59
VAS Back pain 1 year	Patients with available scores	111	30	36	177	
	Improvement above the MCID	62 (56%)	12 (40%)	18 (50%)	92 (52%)	0.29
VAS Back pain 2 years	Patients with available scores	92	21	33	146	
	Improvement above the MCID	51 (55%)	8 (38%)	16 (48%)	75 (51%)	0.35
VAS leg pain 1 year	Patients with available scores	107	27	38	172	
	Improvement above the MCID	77 (72%)	12 (44%)	28 (74%)	117 (68%)	0.02
VAS leg pain 2 years	Patients with available scores	90	17	34	141	
	Improvement above the MCID	60 (67%)	8 (47%)	22 (65%)	90 (64%)	0.30

ODI—Oswestry Disability Index, VASB—visual analogue scale for back pain, VASL—visual analogue scale for leg pain, N—non-smokers, S—smokers, PS—previous smokers.

**Table 5:** Multivariate analyses of potential risk factors for surgical site infection, revision surgery and prolonged hospital stay.

			Confidence Interval		
		Odds Ratio	2.50%	97.50%	*p* Value
Surgical site infection	Past smoking	1.010	0.981	1.040	0.49
	Current smoking	0.985	0.953	1.018	0.37
	Pack years	1.001	1.000	1.001	**0.02**
	Age	1.000	0.999	1.001	0.71
	BMI > 30	1.030	1.004	1.057	0.02
	Diabetes	0.981	0.955	1.006	0.14
Revision surgery	Past smoking	0.978	0.892	1.072	0.63
	Current smoking	1.026	0.923	1.140	0.64
	Pack years	1.001	0.999	1.003	0.16
	Age	1.000	0.998	1.002	0.99
	BMI > 30	1.037	0.956	1.125	0.38
	Diabetes	0.957	0.881	1.039	0.29
length of stay >24 h	Past smoking	0.99	0.81	1.21	0.93
	Current smoking	1.08	0.86	1.36	0.52
	Pack years	1.00	1.00	1.01	0.29
	Age	0.99	0.99	1.00	0.02
	BMI > 30	1.14	0.95	1.36	0.16
	Diabetes	1.02	0.85	1.23	0.81

**Table 6:** Multivariate analyses of potential predictors of improvement exceeding MCID in each patient’s reported outcome measures.

			Confidence Interval		
		Odds Ratio	2.50%	97.50%	*p* Value
ODI 1 year	Past smoking	0.863	0.705	1.056	0.16
	Current smoking	0.764	0.606	0.964	0.02
	Pack years	1.002	0.998	1.007	0.28
	Age	0.995	0.991	1.000	0.04
	BMI > 30	0.969	0.811	1.159	0.73
	Diabetes	1.023	0.853	1.227	0.80
ODI 2 years	Past smoking	0.960	0.784	1.175	0.69
	Current smoking	0.854	0.678	1.077	0.19
	Pack years	1.001	0.997	1.006	0.59
	Age	0.998	0.994	1.002	0.37
	BMI > 30	0.963	0.806	1.151	0.68
	Diabetes	0.861	0.718	1.033	0.11
VAS Back pain 1 year	Past smoking	0.905	0.738	1.111	0.34
	Current smoking	0.817	0.646	1.034	0.90
	Pack years	1.001	0.997	1.006	0.62
	Age	1.000	0.995	1.004	0.93
	BMI > 30	1.003	0.837	1.202	0.97
	Diabetes	1.139	0.947	1.369	0.17
VAS Back pain 2 years	Past smoking	0.963	0.788	1.177	0.72
	Current smoking	0.815	0.647	1.026	0.08
	Pack years	1.000	0.996	1.005	0.96
	Age	0.999	0.995	1.003	0.70
	BMI > 30	1.009	0.845	1.204	0.92
	Diabetes	1.112	0.928	1.331	0.25
VAS leg pain 1 year	Past smoking	1.081	0.891	1.311	0.43
	Current smoking	0.798	0.639	0.996	0.04
	Pack years	0.998	0.994	1.002	0.31
	Age	0.996	0.992	1.000	0.06
	BMI > 30	1.063	0.896	1.261	0.48
	Diabetes	1.052	0.884	1.251	0.57
VAS leg pain 2 years	Past smoking	0.963	0.788	1.177	0.71
	Current smoking	0.815	0.647	1.026	0.08
	Pack years	1.000	0.996	1.005	0.96
	Age	0.999	0.995	1.003	0.70
	BMI > 30	1.009	0.845	1.204	0.92
	Diabetes	1.112	0.928	1.331	0.25
